# Tumor-associated macrophages correlate with better outcome in SHH medulloblastoma

**DOI:** 10.3389/fonc.2025.1557313

**Published:** 2025-04-15

**Authors:** Jin Zhang, Shuting Li, Yuan Wang, Jingjing Liu, Yan Liu, Xiaojun Gong, Yanling Sun, Liming Sun, Zhigang Li, Tianyou Wang, Shuxu Du, Wanshui Wu

**Affiliations:** ^1^ Department of Pediatrics, Beijing Shijitan Hospital, Capital Medical University, Beijing, China; ^2^ Hematologic Disease Laboratory, Hematology Center, Beijing Key Laboratory of Pediatric Hematology Oncology, National Key Discipline of Pediatrics (Capital Medical University), Key Laboratory of Major Disease in Children, Ministry of Education, Beijing Pediatric Research Institute, Beijing Children’s Hospital, Capital Medical University, National Center for Children’s Health, Beijing, China; ^3^ Hematology Center, Beijing Key Laboratory of Pediatric Hematology Oncology, National Key Discipline of Pediatrics (Capital Medical University), Key Laboratory of Major Disease in Children, Ministry of Education, Beijing Children’s Hospital, Capital Medical University, National Center for Children’s Health, Beijing, China

**Keywords:** medulloblastoma, macrophage, phenotype, age, prognosis

## Abstract

**Objective:**

Tumor-associated macrophages (TAMs) constitute a significant proportion of the immune cell population within brain tumors. The polarization of macrophages exerts an important influence on the tumor microenvironment (TME). Nevertheless, the specific role of TAMs in sonic hedgehog (SHH) medulloblastoma remains unclear. To investigate the polarization characteristics and effects of TAMs in SHH medulloblastoma, we evaluated the infiltration of M1 and M2 macrophages in SHH medulloblastoma tissues and analyzed the correlation between TAMs recruitment and the clinical outcome of SHH medulloblastoma patients.

**Methods:**

We enrolled a total of 42 patients diagnosed with SHH medulloblastoma. Using multiple immunofluorescence staining on paraffin-embedded sections, we detected the activated phenotype (M1/M2) by monoclonal antibodies for CD68, HLA-DR and CD163. Subsequently, we analyzed the correlation between TAMs and clinical characteristics as well as prognostic factors.

**Results:**

The median age of 42 patients (31 boys, 11 girls) was 5.3 years (range: 0.8-15.1 years). All patients had confirmed pathological types, including 4 cases of classic medulloblastoma (CMB), 33 cases of desmoplastic/nodular medulloblastoma (DNMB), 3 cases of medulloblastoma with extensive nodularity (MBEN), and 2 cases of large-cell/anaplastic medulloblastoma (LCA). Thirteen cases presented with metastasis at diagnosis, while twenty-nine cases were without metastasis. Four cases had high-risk genetic abnormalities. Different proportions of macrophages were found in the collected medulloblastoma tissues, and large amounts of CD68^+^HLA-DR^+^CD163^+^ cells were found. The study revealed that M_total_ (total macrophages) and M_mix_ (CD68^+^HLA-DR^+^CD163^+^ cells) were significantly higher in group of patients <5 years old (*P <* 0.05), and M_total_ in non-metastatic group were significantly higher than that in metastatic group (*P* = 0.043). M2 macrophages in CMB group were significantly higher than that in DNMB/MBEN group (*P* = 0.036), M1 macrophages were significantly higher in children without high-risk genetic abnormalities (*P* = 0.007). Five-year PFS was significantly poorer in patients ≥5 years old and metastatic group (*P* < 0.05). High M_total_ group had a better 5-year PFS (*P* = 0.000), whereas high M2 group had both better 5-year PFS and OS (*P =* 0.001, *P =* 0.001). Multivariate analysis showed that M_total_ and M2 macrophages were independent prognostic factors for 5-year PFS, and M2 macrophages were an independent prognostic factor for 5-year OS.

**Conclusion:**

The increase in total macrophages and M2 macrophages predicts a better outcome of SHH medulloblastoma. TAMs especially M2 macrophages might be a therapeutic target for SHH medulloblastoma.

## Introduction

Medulloblastoma is the most common malignancy of the central nervous system in childhood, which presents with a series of molecular diversity. The SHH subgroup of medulloblastoma accounts for about 30% of all molecular subtypes and is the most deeply studied type at present (1). Tumor-associated macrophages (TAMs) are the main immune cells in brain tumor tissues and macrophage polarization plays an important role in the progression of brain tumors (2, 3). It is generally recognized that M1 and M2 indicate macrophages lie at opposite ends of the polarization process, and M1 macrophages exert proinflammatory anti-tumor functions by direct killing of tumor cells, tumor antigen presentation, and the promotion of adaptive immuneresponses, while M2 macrophages exert pro-tumoral effects by promoting tumor growth, angiogenesis and metastasis (4, 5). In brain tumors, M1 macrophages can directly phagocytose or kill tumor cells through antibody-dependent cytotoxicity and cause vascular damage and tumor necrosis by generating a variety of cytokines, such as IL-1, IL-6, IL-12, IL-23, TNF-α, NO and ROS (6, 7). Also, M1 macrophages can recruit Th1 cells to the tumor site through the secretion of the chemokines CXCL9/10. M2 macrophages can cause immune suppression and tumor progression by secreting a wide array of cytokines and growth factors such as IL-4, IL-5, IL-10, IL-13, TGF-β and VEGF (6, 7). Additionally, M2 can help recruit Th2 cells that release IL-4, IL-5 and IL-10.

Several studies have shown that SHH medulloblastoma has a significantly higher infiltration of TAMs (8–10). However, the role of TAMs in SHH medulloblastoma remains controversial. Lee C et al. (11) showed that the recruitment of M1 macrophages correlated with worse outcome in SHH medulloblastoma. Bockmayr M et al. (9) found no significant association between survival and macrophages in any subgroups. Therefore, the role of TAMs in SHH medulloblastoma is still unclear and needs further study.

Our previous research demonstrated that an increase of the total macrophages and M1 macrophages was associated with better outcomes in all medulloblastoma patients (10). Notably, the SHH subgroup exhibited a specific elevation in both total macrophages and M2 macrophages. To further elucidate these findings, we conducted an analysis of the correlation between TAMs and the clinical characteristics and outcomes of pediatric patients with SHH medulloblastoma.

## Materials and methods

### Patients and samples

This study was conducted as a retrospective analysis, and the testing of patient samples was performed on a voluntary basis. All participants were newly diagnosed and underwent routine treatment and were followed up regularly. Based on the formula below for sample size calculation in Cox regression model, the calculated sample size is 39.


$$N=\frac{{\left(Z_{1-\alpha /2}+Z_{1-\beta }\right)}^{2}}{P\left(1-R^{2}\right)\sigma^{2}B^{2}}$$

A total of 42 children with SHH medulloblastoma were included who underwent surgical resection between 2015 and 2020. Treatment was performed according to the German Society of Pediatric Oncology and Hematology (GPOH) Protocol HIT-2000 (12, 13). Thirty-seven patients received chemotherapy and radiotherapy after surgery. Five patients aged < 3 years at diagnosis only received chemotherapy. Patient information such as gender, age of onset, pathological subtype, molecular subtype, tumor stage and treatment outcome were collected through medical record review by researchers not involved in conducting the experiments. This study was approved by the Ethics Committee of Beijing Shijitan Hospital, and informed consent was obtained from all of the patients and/or their parents.

### Immunofluorescence staining

Paraffin sections with a thickness of 5 µm were prepared and subsequently stained for CD68, HLA-DR, and CD163 using a multiplex immunofluorescence technique. Multiplex immunofluorescence staining was performed using PANO 4-plex IHC kit (catalog number: 0001100020, Panovue, Beijing, China). The kit included monochromatic fluorescent dyes (Opal 520, Opal 570, Opal 650 and DAPI), a signal amplification reaction buffer, and a secondary antibody (polymer-conjugated horseradish peroxidase (HRP) anti-mouse/rabbit IgG). The primary antibodies used were rabbit anti-human CD68 (catalog number: 0030300020), rabbit anti-human HLA-DR (catalog number: 0036500020) and rabbit anti-human CD163 (catalog number: 0026200025), all sourced from Panovue, Beijing, China. All operations were conducted in full compliance with the specified instructions. The detailed experimental procedures were as follows. First, the slides were placed in a thermostatic oven at 56 °C for 2 h. Subsequently, the slides underwent deparaffinization in xylene and dehydration through a graded ethanol series. Antigen retrieval was performed using microwave heating in an alkaline antigen retrieval solution (catalog number: 0019020500). The slides were then blocked with an antibody blocking solution (catalog number: 0018001030), followed by incubation with primary antibodies at room temperature for 1 h. Following this, the slides were incubated with the secondary antibody for 10 minutes. Subsequently, each antigen was labeled with distinct fluorophores, and the immunofluorescence signal was amplified using tyramide signal amplification (TSA). Microwave antigen retrieval was repeated after each staining cycle. The labeling sequence and antibody-fluorophore combinations were as follows: CD68 (1:300) with Opal 520 (1:100), CD163 (1:200) with Opal 570 (1:100), and HLA-DR (1:300) with Opal 650 (1:100). Finally, the nuclei were stained with DAPI (1:100) and the slides were sealed with anti-fluorescence quencher.

### Digital image analysis

The stained slides were scanned using the Vectra-Polaris Automated Quantitative Pathology Imaging System (Akoya Biosciences, USA). Firstly, full-scan images were obtained, and then five regions (image size: 930µm×700µm) were randomly selected from the whole areas for detailed analysis. The quantity and proportion of cells exhibiting fluorescent signal were calculated using the inForm image analysis software (version 2.4.0). Finally, the average proportions of positive cells in the five regions were calculated. Same as our previous study (7), CD68^+^ cells were designated as the total macrophages (M_total_), CD68^+^HLA-DR^+^CD163^−^ cells were defined as M1 macrophages, CD68^+^CD163^+^HLA-DR^−^ cells were defined as M2 macrophages, and CD68^+^HLA-DR^+^CD163^+^ cells were defined as mixed phenotype macrophages M_mix_.

### Statistical analysis

SPSS 24.0 statistical software was used for analysis, and a *P* value < 0.05 was considered statistically significant. The variables disobeying normal distribution were presented by the median (range), and the Mann-Whitney test was used for comparison between different groups. Kaplan-Meier method was used to analyze the survival rate and univariate survival analyses, Log-rank test was used to compare the difference in survival rate between different groups. Multivariate survival analyses were performed using the Cox regression model, and the hazard ratio (HR) and 95% confidence interval (CI) were calculated. Progression-free survival (PFS) was defined as the time from the date of surgery to date of progression. Overall survival (OS) was defined as the time from the date of surgery to date of death or the last follow-up. The last follow-up time was October 31st, 2024. Two members of the research team operated the SPSS software independently and verified the consistency of the results to ensure the accuracy and reliability of the statistical analysis.

## Results

### Patient characteristics

A total of 42 children (31 boys, 11 girls) with SHH medulloblastoma were enrolled in this study, and the clinical characteristics of all patients were shown in **Table 1**. The median age at diagnosis was 5.3 years (0.8-15.1 years). These patients included 4 cases of CMB, 33 cases of DNMB, 3 cases of MBEN and 2 cases of LCA. In the patients aged <5 years, 2 cases were diagnosed with LCA, while the remaining cases were diagnosed with DNMB/MBEN. There were 13 and 29 cases with or without metastasis at diagnosis respectively. Four cases presented with high-risk genetic abnormalities, including 3 with TP53 mutation and 1 with MYC amplification. The median follow-up time was 52 months (14-82 months). The 5-year PFS and OS were 58.7% ± 7.9% and 78.9% ± 6.2%, respectively.

**Table 1:** Relationship of clinical characteristics and TAMs in SHH subgroup.

Characteristics	Number (%)	*P(*M_total_)	*P(*M1)	*P(*M2)	*P(*M_mix_)
Age (year)		0.001	0.606	0.056	0.011
<5	21(50)				
≥5	21(50)				
Gender		0.577	0.338	0.637	0.112
boy	31(74)				
girl	11(26)				
Pathological type		0.391	0.499	0.036	0.259
CMB	4(10)				
DNMB	33(78)				
MBEN	3(7)				
LCA	2(5)				
Metastasis		0.043	0.796	0.242	0.236
yes	13(31)				
no	29(69)				
High-risk gene mutation		0.966	0.007	0.391	0.247
yes	4(9)				
no	38(91)				

CMB, classic medulloblastoma; DNMB, desmoplastic/nodular medulloblastoma; MBEN, medulloblastoma with extensive nodularity; LCA, large-cell/anaplastic medulloblastoma.

### Association between TAMs and patient characteristics

**Figure 1:**
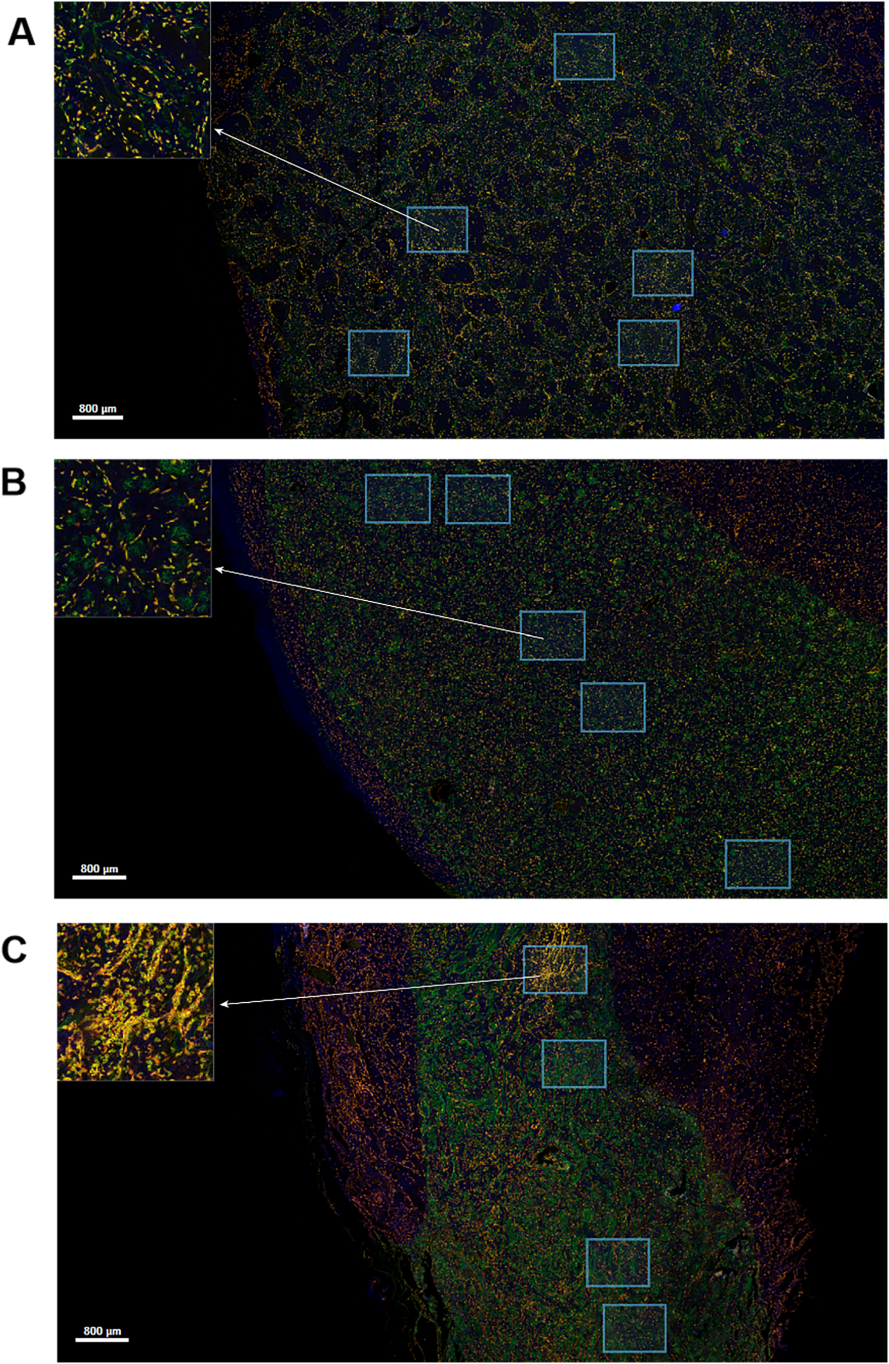
Multiplex immunofluorescence staining image for CD68, CD163 and HLA-DR markers. Green staining is indicative of CD68-positive cells. Yellow staining is indicative of CD163-positive cells. Red staining is indicative of HLA-DR-positive cells. Blue staining is indicative of all cells. The regions indicated by the arrows are enlarged by the software. Scale bar, 800μm. **(A)** Image of a patient without high-risk factors. **(B)** Image of a patient with TP53 mutation. **(C)** Image of a patient with MYC amplification.

**Figure 2:**
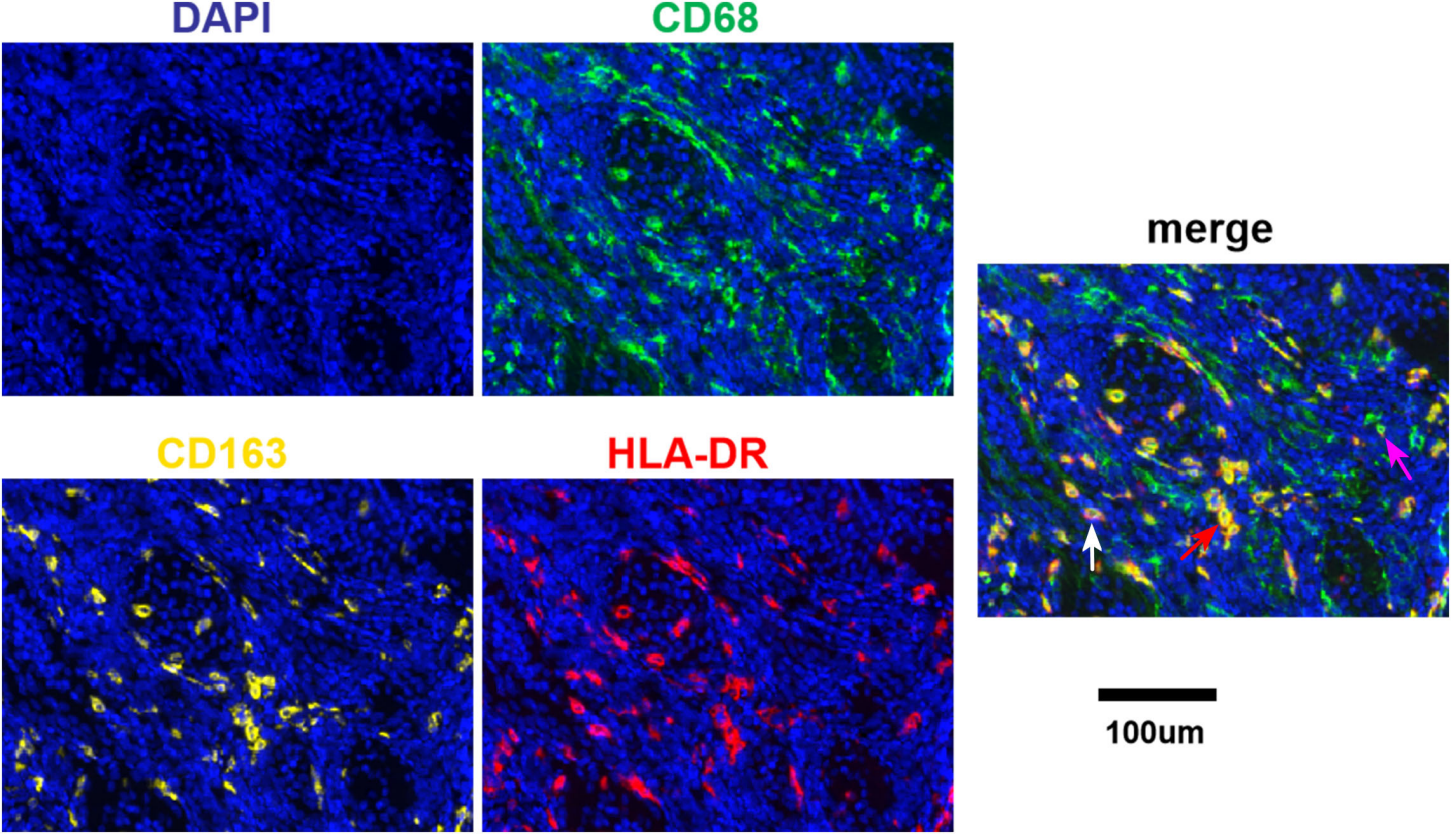
Fluorescence signal splitting and merging images. Green staining is indicative of CD68-positive cells. Yellow staining is indicative of CD163-positive cells. Red staining is indicative of HLA-DR-positive cells. Blue staining is indicative of all cells. The white arrow indicates M1. The purple arrow indicates M2. The red arrow indicates M_mix_. Scale bar, 100μm.

Different degrees of macrophage infiltration were observed in the tissue samples of all patients. The results showed that the median proportion of M_total_ was 7.35% (3.07%-17.39%), M1 was 1.50% (0.26%-3.15%), M2 was 0.60% (0.02%-1.88%) and M_mix_ was 2.54% (0.51%-7.53%). The representative immunofluorescence images are shown in **Figures 1A–C**, **2**. A distinct hotspot area for macrophages was observed in a single case of MYC-amplified medulloblastoma (**Figure 1C**).

We compared the proportion of different types of TAMs in patients with different clinical characteristics, as shown in **Table 1**. It was found that M_total_ (*P* = 0.001) and M_mix_ (*P* = 0.011) were significantly higher in children < 5 years old than those in children ≥ 5 years old. M_total_ was significantly higher in non-metastatic patients than that in metastatic group (*P* = 0.043). M1 was significantly higher in patients without high-risk gene mutations than that in high-risk gene mutation group (*P* = 0.007). M2 in CMB group was significantly higher than that in DNMB/MBEN group (*P* = 0.036). There was no significant relationship between other clinical characteristics and infiltrating macrophages (*P* > 0.05).

### Association between TAMs and survival in SHH medulloblastoma

The correlation between different types of macrophages, clinical characteristics and patient prognosis was further analyzed. According to the median percentage of macrophages (M_total_: 7.35%, M1: 1.50%, M2: 0.60%, M_mix_: 2.54%), the children were divided into two groups: the high percentage group and the low percentage group. As shown in **Table 2**, the results showed that 5-year PFS was significantly higher in the high M_total_ (*P* = 0.000) and M2 (*P* = 0.001) groups, and < 5 years old group (*P* = 0.008) and non-metastatic group (*P* = 0.017). Five-year OS was significantly higher in the high M2 group (*P* = 0.001). Furthermore, age and metastasis were prognostic factors affecting the 5-year PFS. There was no relationship of other macrophages and clinical features with 5-year PFS or OS (*P* > 0.05).

**Table 2:** Univariate analyses of clinical variables associated with PFS and OS.

Clinical Factor		M_total_	M1	M2	M_mix_	Age	Gender	Metastasis	Pathological type	High-risk gene mutation
*P*	PFS	0.000	0.385	0.001	0.065	0.008	0.725	0.017	0.848	0.389
	OS	0.054	0.878	0.001	0.767	0.284	0.721	0.092	0.960	0.244

Statistically significant factors for 5-year PFS were incorporated into the Cox regression model, as shown in **Table 3**. Multivariate analysis showed that M_total_ (*P* = 0.045, HR = 0.289, 95% CI = 0.086-0.971) and M2 (*P* = 0.032, HR = 0.313, 95% CI = 0.108-0.907) were independent prognostic factors affecting the 5-year PFS and they were protective factors for the prognosis of children. In the Cox regression model for 5-year OS, as shown in **Table 4**, M2 (*P* = 0.025, HR = 0.153, 95% CI = 0.030-0.786) was an independent prognostic factor and a protective factor for the prognosis of children.

**Table 3:** Multivariate analysis of clinical variables associated with PFS.

Clinical Factor	*P*	HR	95% CI
M_total_	0.045	0.289	0.086-0.971
M2	0.032	0.313	0.108-0.907
Age	0.828	0.138	0.354-3.657
Metastasis	0.099	2.305	0.854-6.220

HR, hazard ratio; CI, confidence interval.

**Table 4:** Multivariate analysis of clinical variables associated with OS.

Clinical Factor	*P*	HR	95% CI
M_total_	0.740	0.785	0.187-3.288
M2	0.025	0.153	0.030-0.786
Metastasis	0.311	1.823	0.571-5.827

HR, hazard ratio; CI, confidence interval.

## Discussion

At present, the role of TAMs in SHH medulloblastoma remains unclear. Our previous research revealed that the total macrophages and M2 macrophages are specifically increased in SHH subgroup (10), so we analyzed the TAMs characteristics in SHH medulloblastoma for further investigation. In this study, we observed that the total macrophages and M2 macrophages were independent factors affecting the prognosis of children with SHH medulloblastoma, and the increase of the total macrophages predicted a better 5-year PFS, while the increase of M2 macrophages predicted a better 5-year PFS and OS.

TAMs infiltration is most abundant in SHH medulloblastoma (14). Given the plasticity of macrophages, the selection of effective TAMs markers is crucial. According to literature reports, CD68, HLA-DR and CD163 are currently mature phenotypic markers for TAMs (15). In our study, we used CD68, CD163 and HLA-DR to co-label macrophages and stained all three antigens on a single section, which avoided the omission of traditional immunohistochemistry in co-localization, making the results more accurate and also saving the amount of specimens.

TAMs are the main component of the tumor microenvironment (TME), usually accounting for 30% of the tumor volume (16). In glioblastoma, TAMs represent up to one half of the tumor mass cells (6). In our study, the proportion of the total macrophages was 3.07%-17.39%, which is similar to Lee C’s (11) study (approximately 3%-12%). However, TAMs in medulloblastoma appeared to be less abundant compared to those in glioma. It is reported that TAMs are mainly composed of different proportions of both resident brain microglia and infiltrating macrophages (17, 18). Maximov V et al. (19) showed monocyte recruitment as the main source of TAMs (75%) in SHH medulloblastoma, and resident brain microglia constituted a relatively small proportion (20%). A limitation of our study is that it did not distinguish between microglia and monocyte derived macrophages. We intend to supplement this aspect in subsequent research.

In this study, we showed that the increase in total macrophages predicted a better outcome, and which was consistent with our previous conclusions. It also demonstrated that the total macrophages were higher in patients under 5 years old and patients without metastasis in this study. Maximov V et al. (19) also demonstrated that TAMs in SHH medulloblastoma exhibited cytotoxic activities against tumor cells and the presence of macrophages may positively contribute to patient survival outcomes. A potential mechanism involved the recruitment of monocytes from the bloodstream to tumor tissue via a gradient of CCL2 cytokine secreted by TME, followed by their differentiation into macrophages. However, their study did not distinguish between M1 and M2, and the precise anti-tumor mechanism remains to be elucidated. M1 macrophages were higher in patients without high-risk gene mutations, which was consistent with the antitumor effect of M1 macrophages (20). In addition, a distinct hotspot area for macrophage aggregation was observed in a single case of MYC-amplified medulloblastoma. Nevertheless, given the limited sample size, the specific findings require further validation. A recent study demonstrated that both M1 and M2 markers, secreted by exosomes, were increased in SHH TP53-mutated and MYC-amplified medulloblastomas (21). However, in this study, we did not observe a significant association between M1 macrophages and the prognosis of SHH medulloblastoma patients. In contrast, Lee C et al. (11) reported that an increase in M1 macrophages correlated with poorer outcomes in SHH medulloblastoma. It was noteworthy that the increase of M2 macrophages indicated a better prognosis in SHH subgroup, which seemed to contradict with the tumor-promoting effect of M2 macrophages, but it also demonstrated the heterogeneity and complexity of macrophages. Wu KS et al. (22) reached the same conclusion, observing that M2 macrophages were related to the good outcome of SHH medulloblastoma, and a high expression of CCL2 was observed concurrently with increased M2 macrophage infiltration. In Lee C’s study, no significant association between M2 macrophages and the survival of SHH medulloblastoma was observed (11). The variability could potentially arise from the heterogeneity in different pathological and granular molecular subtypes. The majority of SHH medulloblastoma cases present with DNMB subtype. In this study, M2 macrophages increased in the CMB group. However, further investigation is warranted due to the limited number of CMB cases which may constrain the generalizability of our findings. Furthermore, we are particularly interested in LCA cases which have the worst prognosis of all medulloblastomas, but the sample size is also insufficient. Additional substructure has been described dividing SHH medulloblastoma into four more granular molecular groups (SHH-α, β, γ, δ), which are enriched for specific genomic abnormalities and have distinct clinical associations (23). It suggests that we need to make a more detailed division of SHH medulloblastoma.

Currently, the underlying anti-tumor mechanism of M2 macrophages remains an enigma. Ray A et al. (24) reported that early ‘M2-like’ TAMs depletion led to an indirect loss of a key anti-tumor network of NK cells, conventional type I dendritic cells and CD8 T cells, and they were the primary producers of CXCL9, which differentially attracted activated CD8 T cells, affecting the tumor control by CD8 T cells. Additionally, de Santis JO et al. (21) showed that the proportion of M2 macrophages was decreased in patients presented with tumor dissemination. Dang MT et al. (17) described that the signature of M2 macrophages is higher in WNT tumors. These findings might indicate the positive effects of M2 macrophages from an indirect perspective. However, research on the specific mechanisms remains insufficient and warrants further investigation.

However, M1 and M2 cannot fully represent the phenotypic diversity and comprehensive function of TAMs. The polarization of macrophages to M2 appears to be an oversimplification, and some researchers have further categorized M2 macrophages into subtypes including M2a, M2b, M2c, and M2d (25). TAMs are in a constant transition state between M1 and M2 forms. In this study, a large number of mixed phenotype cells were observed, indicating that most macrophages are in a mixed state of M1/M2. Although our study did not identify a relationship between M_mix_ with patient prognosis, these cells are considered to be of critical importance. One study showed that the mixed M1/M2 macrophage phenotype was associated with tumor aggressiveness (26). Moreover, tumor reduction was correlated with the proportion of the mixed M1/M2 phenotype (27). Macrophages exhibit strong plasticity, and the therapeutic strategy targeting TAMs is to explore approaches to reprogram these cells towards a more beneficial phenotype.

Age and metastasis are known prognostic factors for SHH medulloblastoma (28). Our study showed that age ≥ 5 years old and metastasis indicated worse 5-year PFS of children, and disease progression and death mostly occurred in children 5 years of age or older, suggesting that more attention and research should be paid to these patients. Meanwhile, in children ≤ 5 years old, two distinct subtypes of infant SHH (iSHH), called iSHH-I and iSHH-II, have been identified (1), and the prognosis of the two is significantly different. It is reported that patients with iSHH-II exhibited a 5-year PFS of 75.4%, whereas those with iSHH-I had a 5-year PFS of 27.8% (29). In our study, the proportion of patients with metastasis was 13/42 (31%), which represents a notably high rate. Patients with metastasis exhibited a greater propensity to participate in this study. This may introduce potential bias into our findings. Wu KS et al. reported that tumor metastasis occurred in both SHHα (16.7%) and SHHβ (33.3%), but not in SHHγ (22). However, we lack data on the granular molecular groups of these patients, which could include a higher proportion of SHHβ cases. These findings suggest that more accurate and individualized treatment measures should be taken according to the age and molecular characteristics of children.

Studies using animal models have shown that TAMs present dynamic changes accompanied by treatment and disease progression (17, 30). The limitation of this study is that the results can only reflect the state of TAMs before treatment. Our team is currently endeavoring to conduct dynamic monitoring of cerebrospinal fluid cytokines to address this limitation. In addition, the sample size needs to be further increased to make more detailed division of SHH medulloblastoma to discover more features.

## Conclusion

The increase in total macrophages and M2 macrophages predicted a better outcome of children in SHH medulloblastoma, and M2 macrophages might be taken as a therapeutic target for SHH medulloblastoma.

## Data Availability

The original contributions presented in the study are included in the article/supplementary material. Further inquiries can be directed to the corresponding authors.

## References

[1] Northcott PA Robinson GW Kratz CP Mabbott DJ Pomeroy SL Clifford SC. Medulloblastoma. Nat Rev Dis Primers. (2019) 5:11. doi: 10.1038/s41572-019-0063-6 30765705

[2] Guadagno E Presta I Maisano D Donato A Pirrone CK Cardillo G. Role of macrophages in brain tumor growth and progression. Int J Mol Sci. (2018) 19:1005. doi: 10.3390/ijms19041005 29584702 PMC5979398

[3] Klemm F Maas RR Bowman RL Kornete M Soukup K Nassiri S. Interrogation of the microenvironmental landscape in brain tumors reveals disease-specific alterations of immune cells. Cell. (2020) 181:1643–1660.e17. doi: 10.1016/j.cell.2020.05.007 32470396 PMC8558904

[4] Zhang Q Sioud M. Tumor-associated macrophage subsets: shaping polarization and targeting. Int J Mol Sci. (2023) 24:7493. doi: 10.3390/ijms24087493 37108657 PMC10138703

[5] Vitale C Bottino C Castriconi R. Monocyte and macrophage in neuroblastoma: blocking their pro-tumoral functions and strengthening their crosstalk with natural killer cells. Cells. (2023) 12:885. doi: 10.3390/cells12060885 36980226 PMC10047506

[6] Caverzán MD Beaugé L Oliveda PM Cesca González B Bühler EM Ibarra LE. Exploring monocytes-macrophages in immune microenvironment of glioblastoma for the design of novel therapeutic strategies. Brain Sci. (2023) 13:542. doi: 10.3390/brainsci13040542 37190507 PMC10136702

[7] Wang JC Li SL Lan YJ Liu X Li WB. Glioma-associated macrophages: unraveling their dual role in the microenvironment and therapeutic implications. Curr Med. (2024) 3:4. doi: 10.1007/s44194-024-00031-y

[8] Margol AS Robison NJ Gnanachandran J Hung LT Kennedy RJ Vali M. Tumor-associated macrophages in SHH subgroup of medulloblastomas. Clin Cancer Res. (2015) 21:1457–65. doi: 10.1158/1078-0432.CCR-14-1144 PMC7654723 25344580

[9] Bockmayr M Mohme M Klauschen F Winkler B Budczies J Rutkowski S. Subgroup-specific immune and stromal microenvironment in medulloblastoma. Oncoimmunology. (2018) 7:e1462430. doi: 10.1080/2162402X.2018.1462430 30228931 PMC6140816

[10] Zhang J Yuan X Wang Y Liu J Li Z Li S. Tumor-associated macrophages correlate with prognosis in medulloblastoma. Front Oncol. (2022) 12:893132. doi: 10.3389/fonc.2022.893132 35860588 PMC9289152

[11] Lee C Lee J Choi SA Kim SK Wang KC Park SH. M1 macrophage recruitment correlates with worse outcome in SHH medulloblastomas. BMC Cancer. (2018) 18:535. doi: 10.1186/s12885-018-4457-8 29739450 PMC5941618

[12] Dietzsch S Placzek F Pietschmann K von Bueren AO Matuschek C Glück A. Evaluation of prognostic factors and role of participation in a randomized trial or a prospective registry in pediatric and adolescent nonmetastatic medulloblastoma - A report from the HIT 2000 trial. Adv Radiat Oncol. (2020) 5:1158–69. doi: 10.1016/j.adro.2020.09.018 PMC7718550 33305077

[13] Mynarek M von Hoff K Pietsch T Ottensmeier H Warmuth-Metz M Bison B. Nonmetastatic medulloblastoma of early childhood: results from the prospective clinical trial HIT-2000 and an extended validation cohort. J Clin Oncol. (2020) 38:2028–40. doi: 10.1200/JCO.19.03057 32330099

[14] Zhu L Yang Y Li H Xu L You H Liu Y. Exosomal MicroRNAs Induce Tumor-associated Macrophages via PPARγ during Tumor Progression in SHH Medulloblastoma. Cancer Lett. (2022) 535:215630. doi: 10.1016/j.canlet.2022.215630 35304257

[15] Rakaee M Busund LR Jamaly S Paulsen EE Richardsen E Andersen S. Prognostic value of macrophage phenotypes in resectable non-small cell lung cancer assessed by multiplex immunohistochemistry. Neoplasia. (2019) 21:282–93. doi: 10.1016/j.neo.2019.01.005 PMC6369140 30743162

[16] Quail DF Joyce JA. The microenvironmental landscape of brain tumors. Cancer Cell. (2017) 31:326–41. doi: 10.1016/j.ccell.2017.02.009 PMC5424263 28292436

[17] Dang MT Gonzalez MV Gaonkar KS Rathi KS Young P Arif S. Macrophages in SHH subgroup medulloblastoma display dynamic heterogeneity that varies with treatment modality. Cell Rep. (2021) 34:108917. doi: 10.1016/j.celrep.2021.108917 33789113 PMC10450591

[18] Khan F Pang L Dunterman M Lesniak MS Heimberger AB Chen P. Macrophages and microglia in glioblastoma: heterogeneity, plasticity, and therapy. J Clin Invest. (2023) 133:e163446. doi: 10.1172/JCI163446 36594466 PMC9797335

[19] Maximov V Chen Z Wei Y Robinson MH Herting CJ Shanmugam NS. Tumour-associated macrophages exhibit anti-tumoural properties in sonic hedgehog medulloblastoma. Nat Commun. (2019) 10:2410. doi: 10.1038/s41467-019-10458-9 31160587 PMC6546707

[20] Huo Y Zhang H Sa L Zheng W He Y Lyu H. M1 polarization enhances the antitumor activity of chimeric antigen receptor macrophages in solid tumors. J Transl Med. (2023) 21:225. doi: 10.1186/s12967-023-04061-2 36978075 PMC10044396

[21] de Santis JO de Sousa GR Queiroz RGP Cândido MF Almeida F de Rezende CP. Immunomodulatory role of exosome-derived content in pediatric medulloblastoma: a molecular subgroup perspective. Hum Cell. (2025) 38:55. doi: 10.1007/s13577-025-01181-3 39960575

[22] Wu KS Sung SY Huang MH Lin YL Chang CC Fang CL. Clinical and molecular features in medulloblastomas subtypes in children in a cohort in Taiwan. Cancers (Basel). (2022) 14:5419. doi: 10.3390/cancers14215419 36358838 PMC9657873

[23] Orr BA. Pathology, diagnostics, and classification of medulloblastoma. Brain Pathol. (2020) 30:664–78. doi: 10.1111/bpa.12837 PMC7317787 32239782

[24] Ray A Hu KH Kersten K Courau T Kuhn NF Zaleta-Linares I. Targeting CD206+ macrophages disrupts the establishment of a key antitumor immune axis. J Exp Med. (2025) 222(1):e20240957. doi: 10.1084/jem.20240957 39601781 PMC11602655

[25] Guo Y Hong W Zhang P Han D Fang Y Tu J. Abnormal polarization of macrophage-like cells in the peripheral blood of patients with glioma. Oncol Lett. (2020) 20:947–54. doi: 10.3892/ol.2020.11602 PMC7285800 32566024

[26] Pe KCS Saetung R Yodsurang V Chaotham C Suppipat K Chanvorachote P. Triple-negative breast cancer influences a mixed M1/M2 macrophage phenotype associated with tumor aggressiveness. PLoS One. (2022) 17(8):e0273044. doi: 10.1371/journal.pone.0273044 35960749 PMC9374254

[27] Eftimie R Barelle C. Mathematical investigation of innate immune responses to lung cancer: the role of macrophages with mixed phenotypes. J Theor Biol. (2021) 524:110739. doi: 10.1016/j.jtbi.2021.110739 33930438

[28] Gupta T Mani S Chatterjee A Dasgupta A Epari S Chinnaswamy G. Risk-stratification for treatment de-intensification in WNT-pathway medulloblastoma: finding the optimal balance between survival and quality of survivorship. Expert Rev Anticancer Ther. (2024) 24:589–98. doi: 10.1080/14737140.2024.2357807 38761170

[29] Robinson GW Rudneva VA Buchhalter I Billups CA Waszak SM Smith KS. Risk-adapted therapy for young children with medulloblastoma (SJYC07): therapeutic and molecular outcomes from a multicentre, phase 2 trial. Lancet Oncol. (2018) 19:768–84. doi: 10.1016/S1470-2045(18)30204-3 PMC6078206 29778738

[30] Wang YN Wang YY Wang J Bai WJ Miao NJ Wang J. Vinblastine resets tumor-associated macrophages toward M1 phenotype and promotes antitumor immune response. J Immunother Cancer. (2023) 11:e007253. doi: 10.1136/jitc-2023-007253 37652576 PMC10476141

